# Atrial rhythm influences catheter tissue contact during radiofrequency catheter ablation of atrial fibrillation: comparison of contact force between sinus rhythm and atrial fibrillation

**DOI:** 10.1007/s00380-015-0763-0

**Published:** 2015-10-26

**Authors:** Hisao Matsuda, Abdul Shokor Parwani, Philipp Attanasio, Martin Huemer, Alexander Wutzler, Florian Blaschke, Wilhelm Haverkamp, Leif-Hendrik Boldt

**Affiliations:** Cardiology, Campus Virchow Klinikum, Charité-Universitätsmedizin Berlin, Augustenburger Platz 1, 13353 Berlin, Germany

**Keywords:** Atrial fibrillation, Catheter ablation, Contact force, Pulmonary vein isolation, Sinus rhythm

## Abstract

Catheter tissue contact force (CF) is an important factor for durable lesion formation during radiofrequency catheter ablation (RFCA) of atrial fibrillation (AF). Since CF varies in the beating heart, atrial rhythm during RFCA may influence CF. A high-density map and RFCA points were obtained in 25 patients undergoing RFCA of AF using a CF-sensing catheter (Tacticath, St. Jude Medical). The operators were blinded to the CF information. Contact type was classified into three categories: constant, variable, and intermittent contact. Average CF and contact type were analyzed according to atrial rhythm (SR vs. AF) and anatomical location. A total of 1364 points (891 points during SR and 473 points during AF) were analyzed. Average CFs showed no significant difference between SR (17.2 ± 11.3 g) and AF (17.2 ± 13.3 g; *p* = 0.99). The distribution of points with an average CF of ≥20 and <10 g also showed no significant difference. However, the distribution of excessive CF (CF ≥40 g) was significantly higher during AF (7.4 %) in comparison with SR (4.2 %; *p* < 0.05). At the anterior area of the right inferior pulmonary vein (RIPV), the average CF during AF was significantly higher than during SR (*p* < 0.05). Constant contact was significantly higher during AF (32.2 %) when compared to SR (9.9 %; *p* < 0.01). Although the average CF was not different between atrial rhythms, constant contact was more often achievable during AF than it was during SR. However, excessive CF also seems to occur more frequently during AF especially at the anterior part of RIPV.

## Introduction

Radiofrequency catheter ablation (RFCA) is an accepted treatment option for symptomatic and drug resistance atrial fibrillation (AF) [1]. The most commonly used ablation strategy is circumferential pulmonary vein isolation (PVI) around the ipsilateral pulmonary vein ostia using irrigated RF current.

Besides power and duration of RF delivery, catheter tissue contact has been shown to be an important factor for lesion size, transmurality and durable lesion formation during RFCA of AF [2]. Insufficient tissue contact may result in incomplete or transient lesion formation, while excessive contact increases the risk of major complications. A novel technology was developed that integrates a contact force (CF) sensor at the tip of an open-irrigated RF catheter [2], which allows continuous real-time and direct measurement of the CF between catheter tip and target tissue.

Some clinical trials [3, 4] showed that CF correlates with clinical outcome in patients with AF undergoing RFCA for AF using CF-sensing catheters. During PVI, an average CF of ≥20 g is an optimal target to avoid insufficient lesion formation, while an average CF of <10 g is correlated with a recurrence of AF [3–5]. However, achievement of good tissue contact is difficult in some cases even despite knowledge of CF. Influences of the anatomical situation [3, 4, 6] upon CF were recently reported. Respiratory condition has also been shown to be a factor influencing CF [7] and may cause left atrial remodeling [8]. Since CF varies in the beating heart, atrial rhythm during the procedure may also influence tissue contact. To our knowledge, a direct comparison of CF according to atrial rhythm during RF for AF using CF-sensing catheters has not yet been published. Therefore, the influence of atrial rhythm upon CF is still quite unclear. The objective of this study was to evaluate the influence of atrial rhythm during ablation upon CF.

## Methods

### Study population

Twenty-five patients with symptomatic, drug-resistant AF who underwent catheter ablation for AF at our center between October 2013 and August 2014 were prospectively enrolled in this study. Written informed consent was obtained from all patients. This study was approved by the Charité Institutional Ethics Committee (EA1/278/14).

### Pulmonary vein isolation and CF measurement

Before procedure, any patient with a left atrium (LA) or left atrial appendage thrombus was excluded using transesophageal echocardiography. The procedure was performed with deep sedation using midazolam and continuous infusion of propofol.

Esophageal temperature was monitored during the whole procedure using a SensiTherm temperature probe (St. Jude Medical). A 6-F decapolar catheter was positioned in the coronary sinus through the left femoral vein. Through the right femoral vein, a steerable 8.5-F long sheath (Agilis, St. Jude Medical) and a non-steerable 8.5-F long sheath (SL0, St. Jude Medical) were introduced into the LA using single transseptal puncture guided by fluoroscopy and pressure monitoring at the needle tip. In one case, transesophageal echocardiography was also used as guidance for the transseptal puncture because of a prior surgical patch closure operation of an atrial septal defect. After transseptal puncture, the CF sensing ablation catheter (TactiCath, St. Jude Medical) was positioned in the left ventricle through the Agilis sheath, and LA angiography was performed during left ventricular high rate pacing (200 beats/min).

LA geometry was acquired using a spiral mapping catheter (IBI Inquiry Optima, St. Jude Medical) in combination with the 3D mapping system (EnSite Navix Velocity, St. Jude Medical). Directly after transseptal puncture, heparin was administered intravenously at a dose of 100 U/kg body weight. Thereafter, activated clotting time (ACT) was checked every 30 min and more heparin was administered when necessary to maintain an ACT of >300 s.

An open-irrigated RF catheter with a CF sensor (Tacticath, St. Jude Medical) was used in all cases. The CF sensing catheter is a steerable 7-F, 3.5-mm tip open irrigated ablation catheter which allows continuous real-time and direct measurement of the CF between the tissue and the catheter-tip [9]. The CF sensor is a triaxial force sensor located between the second and third electrode and has a resolution and sensitivity of 1 g in a bench test [2]. The real-time CF is measured every 50 ms and displayed continuously as the number of grams. The live CF information was not available for operators during the procedure.

For CF analysis, each pulmonary vein was divided into four quadrants: anterior-superior, anterior-inferior, posterior-superior, and posterior-inferior. At each quadrant, at least two points were achieved for CF mapping. When the patients were in AF at the beginning of the procedure, CF mapping was first performed during AF before RFCA. Therefore, the CF sensing catheter was positioned at the designated circumferential lesion line and kept stable without energy delivery. CF parameters were recorded at these points and tagged in the 3D geometry for later reference. After CF mapping during AF, external cardioversion was performed to restore sinus rhythm (SR). RFCA was applied during SR in those patients in which SR could be restored and CF information was recorded during RF ablation at the same points previously analyzed during AF. To ensure comparable conditions, each RF pulse was delivered without dragging manoeuver but with a “point by point” technique.

When the patients were in SR at the beginning of the procedure, CF data were recorded during SR and AF was induced thereafter. Ablation was then performed during AF at the same points previously tagged in the 3-D geometry.

In those patients who could not be cardioverted or in whom AF induction failed, CF information was only available for either SR or AF.

RF energy was delivered in a power-control mode and was limited to 35 W and 60 s for all locations in the LA. When esophageal temperature increased to more than 39 °C, energy delivery was stopped immediately and lower RF energy in the range of 20–25 W was applied.

Isolation of each PV was confirmed by entrance block using the spiral mapping catheter. When PVI was not achieved with circumferential ablation, earliest PV potentials were targeted sequentially until complete isolation was achieved.

When atrial tachycardia remained after the completion of PVI, roof line ablation or mitral isthmus ablation was added to PVI, depending on the mechanism of atrial tachycardia.

### Evaluation of CF parameter

At each mapping or ablation point, 3D anatomical location was recorded. Average CF and the type of tissue contact was evaluated at each point.

According to the average CF, each point was categorized as “low contact” (average CF < 10 g), “sufficient contact” (average CF ≥ 20 g), and “excessive contact” (average CF ≥ 40 g).

**Fig. 1:**
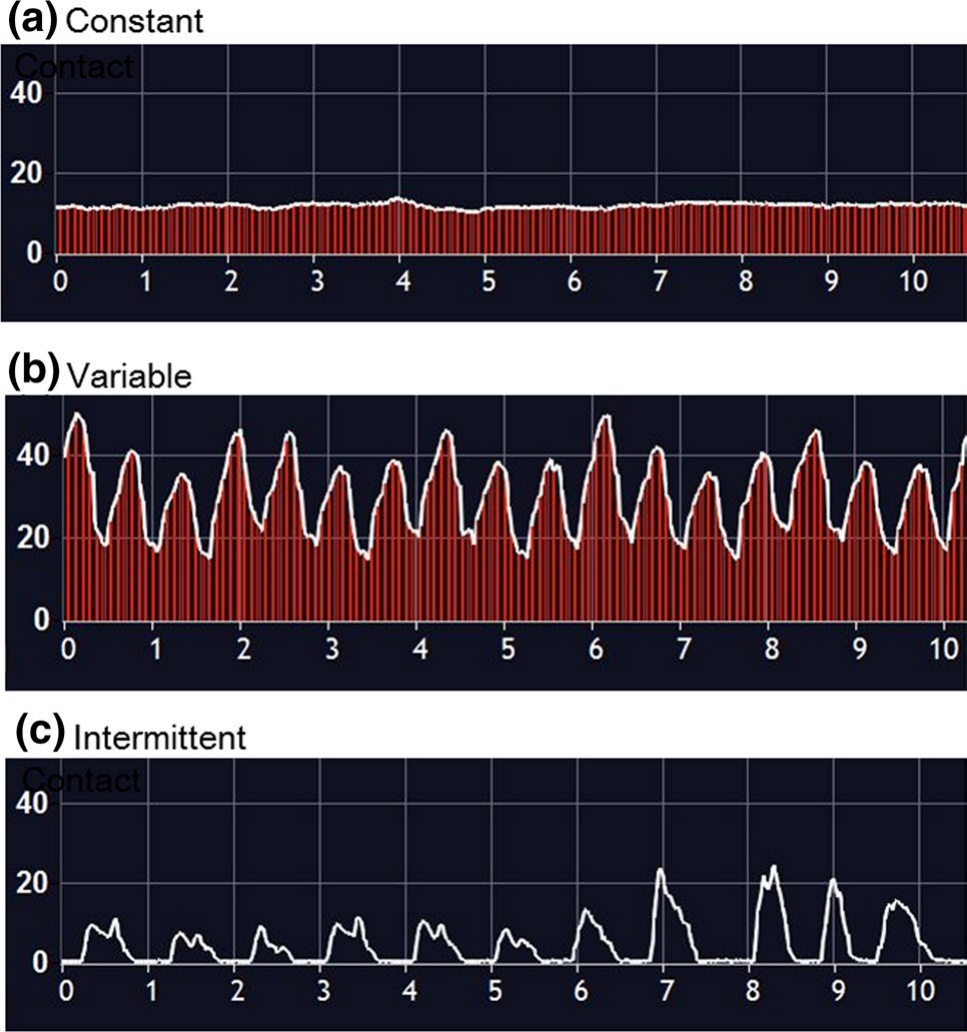
Definition of contact type. **a** Constant contact indicating that the difference of minimal CF and maximal CF was within 10 g. **b** Variable contact indicating that the difference of minimum CF and maximum CF was more than 10 g. **c** Intermittent contact indicating that the contact type with a nadir of 0 g CF (loss of contact) is at least 50 ms, regardless of CF difference between minimum and maximum CF

The type of tissue contact was categorized into three groups (Fig. 1) and was defined as follows:

1. Constant contact: the difference of minimal CF and maximal CF was within 10 g.
2. Variable contact: the difference of minimal CF and maximal CF was more than 10 g.
3. Intermittent contact: regardless of CF difference during mapping or ablation, but with a nadir of 0 g CF (loss of contact) of at least 50 ms.

Average CF and type of contact were analyzed according to atrial rhythms (SR vs. AF).

### Statistical analysis

All continuous variables were expressed as mean ± standard deviation. A Student *t* test and a Chi square test were used for statistical analysis. A two-tailed probability value of *p* < 0.05 was considered to be statistically significant. All statistical analyses were performed using SPSS version 22 (SPSS Inc., Chicago, IL, USA).

## Results

### Clinical characteristics

A total of 1364 points (891 points during SR and 473 points during AF) were acquired and analyzed in 25 patients (age 63.6 ± 7.4 years, 44 % female). Patient characteristics are described in Table 1. CF data during SR could be recorded in 22 patients and during AF in 15 patients. All pulmonary veins were successfully isolated in all of the 25 patients. The numbers of data points in each patient according to atrial rhythms is depicted in Table 2. The procedures were all performed by two experienced operators each who have gathered experience from more than 250 cases of PVI. Also, there was no difference of CF data between the operators.

**Table 1:** Clinical characteristics

Parameter	*N* = 25
Age (years)	63.6 ± 7.4
Male patients	14 (56.0 %)
BMI (kg/m^2^)	27.6 ± 6.6
Persistent AF	10 (40.0 %)
Left atrial dimension (mm)	42.3 ± 5.3
Ejection fraction (%)	54.8 ± 9.7
CHA2DS2-VASc score	2.0 ± 1.3
Hypertension	18 (72.0 %)
Diabetes mellitus	1 (4.0 %)
Vascular disease	7 (28.0 %)
Total procedure time (min)	142 ± 26
Total RF time (min)	30.2 ± 8.0

Continuous variables are shown as mean ± SD

*BMI* body mass index, *AF* atrial fibrillation, *RF* radiofrequency

**Table 2:** The numbers of data points in each patient during SR and AF

Case number	During SR	During AF	Total
1	51	0	51
2	34	12	46
3	36	6	42
4	0	90	90
5	50	0	50
6	3	51	54
7	43	28	71
8	54	0	54
9	67	0	67
10	30	0	30
11	54	22	76
12	16	15	31
13	40	0	40
14	13	20	33
15	44	0	44
16	91	0	91
17	0	46	46
18	67	19	86
19	48	37	85
20	38	15	53
21	4	55	59
22	32	0	32
23	38	0	38
24	0	34	34
25	38	23	61
Total (*N* = 25)	891	473	1364

*SR* sinus rhythm, *AF* atrial fibrillation

### Average contact force during SR and AF

**Fig. 2:**
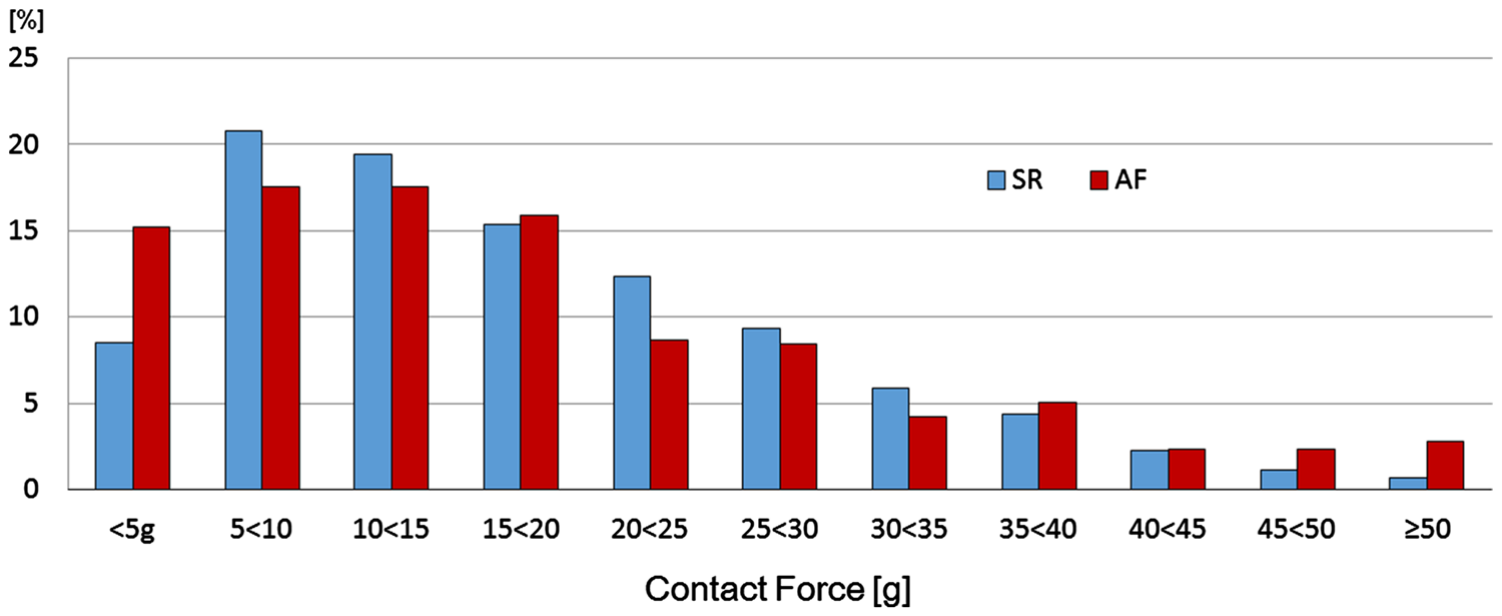
Contact force distribution according to atrial rhythm. Relative distribution of the average contact force. Distribution of contact force of 1364 points in 25 patients. *SR* sinus rhythm, *AF* atrial fibrillation

**Fig. 3:**
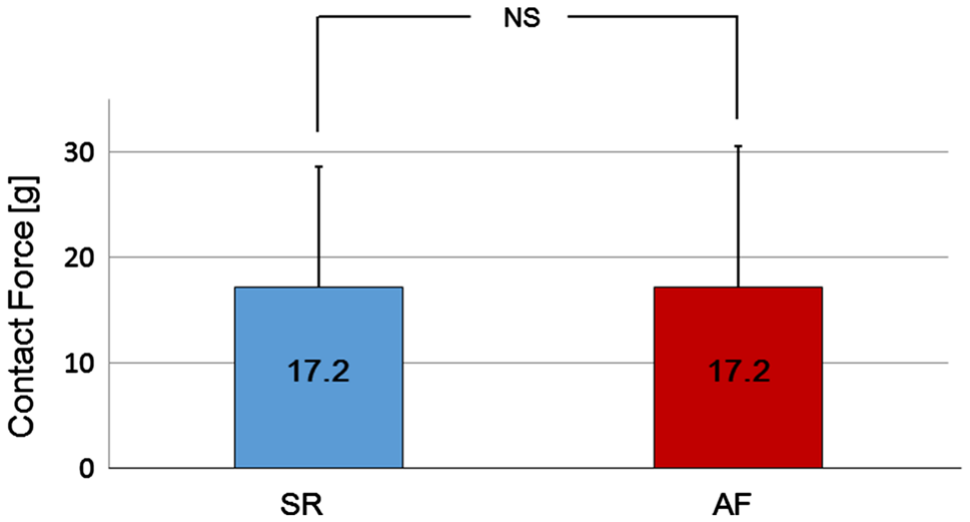
Average contact force during sinus rhythm and atrial fibrillation. The average CF showed no significant difference between atrial rhythms (*p* = 0.99). *SR* sinus rhythm, *AF* atrial fibrillation, *NS* no significant difference

The distribution of average CF showed a high variability in both atrial rhythms (1–90 g during SR, 0–68 g during AF). Peak distribution was 21.5 % in the range of 5–10 g during SR and 17.5 % in the range of 10–15 g during AF. The distribution of CF according to atrial rhythm is shown in Fig. 2. The average CF was 17.2 ± 11.3 g during SR and 17.2 ± 13.3 g during AF. There was no significant difference in a comparison of average CF between SR and AF (*p* = 0.99) (Fig. 3). In a sub-analysis, when we only analyzed those patients in which we could achieve the data points during both atrial rhythms, the average CF still showed no significant difference between atrial rhythms (16.9 ± 11.6 g during SR and 16.0 ± 13.3 g during AF; *p* = 0.30).

**Fig. 4:**
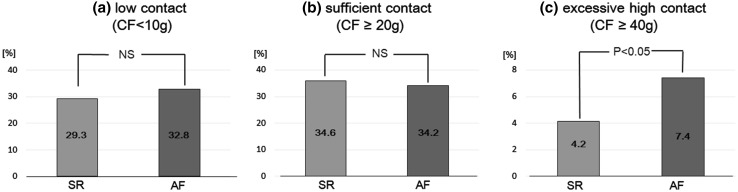
Proportion of each contact force category during SR and AF. The ratio of **a** low contact and **b** sufficient contact showed no significant difference between SR and AF. In contrast to these results, the ratio of **c** excessive contact was significantly higher during AF than it was during SR. *SR* sinus rhythm, *AF* atrial fibrillation, *NS* no significant difference

There was no difference observed in the ratio of low contact (29.3 % during SR vs. 32.8 % during AF; *p* = 0.18) or in the ratio of sufficient contact (36.0 % during SR and 34.2 % during AF; *p* = 0.93) (Fig. 4). In contrast to these results, the ratio of excessive CF was significantly higher during AF (7.4 %) than it was during SR (4.2 %; *p* < 0.05) (Fig. 4).

### Contact type according to atrial rhythm

**Fig. 5:**
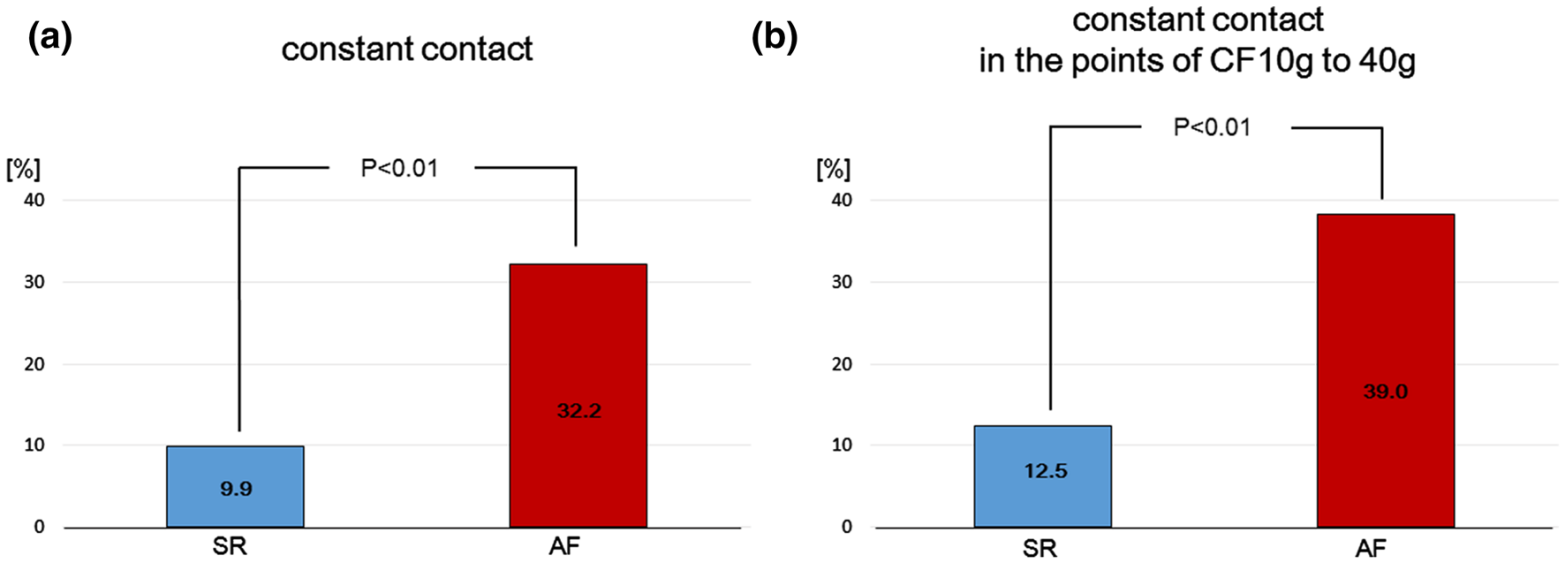
Proportion of constant contact during SR and AF. **a** The constant contact was significantly more achievable during AF than during SR (*p* < 0.01). **b** When only analyzing those points with optimal contact (excluding those points with low or excessively high contact, i.e. CF between 10 and 40 g) the percentage of points with constant contact was still observed to be significantly higher during AF than during SR (*p* < 0.01)

The ratio of contact type during SR was 9.9 % for constant contact, 78.4 % for variable contact, and 11.8 % for intermittent contact. During AF, the ratio of contact type was 33.1, 43.6, and 23.3 %, respectively. The percentage of points with constant contact was significantly higher during AF when compared with SR (*p* < 0.01) (Fig. 5). When we analyzed only those patients in whom we could achieve the data points during both atrial rhythms, the ratio of constant contact was significantly higher during AF (29.0 %) than during SR (8.0 %; *p* < 0.01).

When we analyzed only those points with optimal contact (excluding those points with low or excessively high contact), the percentage of points with constant contact was still significantly higher during AF (39.0 %) than it was during SR (12.5 %; *p* < 0.01) (Fig. 5).

### CF comparison between SR and AF according to anatomical location

**Fig. 6:**
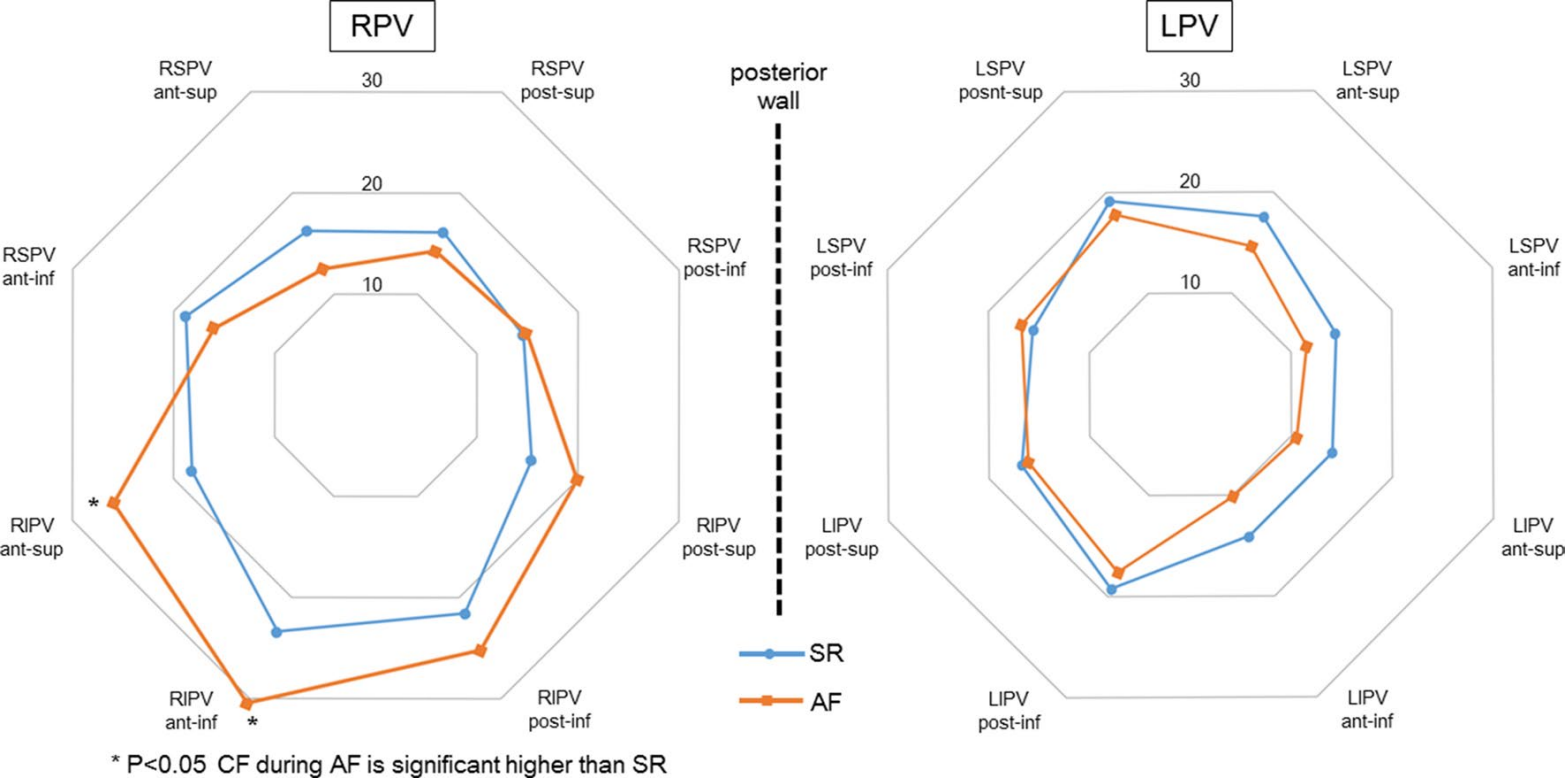
Average CF (g) during SR and AF per each anatomical location. Average CF at the RPV was significantly higher than at the LPV during both atrial rhythms. Lowest CF was observed from anterior-inferior of LSPV and down to anterior-inferior of LIPV (LA ridge area). No significant difference of average CF between atrial rhythms has been shown at the LA ridge area. Although anterior part of RIPV has the tendency to achieve high tissue contact during both atrial rhythms, average CF during AF was significantly higher than SR at the anterior part of RIPV. Except for location at the anterior part of the RIPV, there was no significant difference of average CF seen between atrial rhythms: *RPV* right pulmonary vein, *LPV* left pulmonary vein, *RSPV* right superior pulmonary vein, *RIPV* right inferior pulmonary vein, *LSPV* left superior pulmonary vein, *LIPV* left inferior pulmonary vein, *SR* sinus rhythm, *AF* atrial fibrillation

**Fig. 7:**
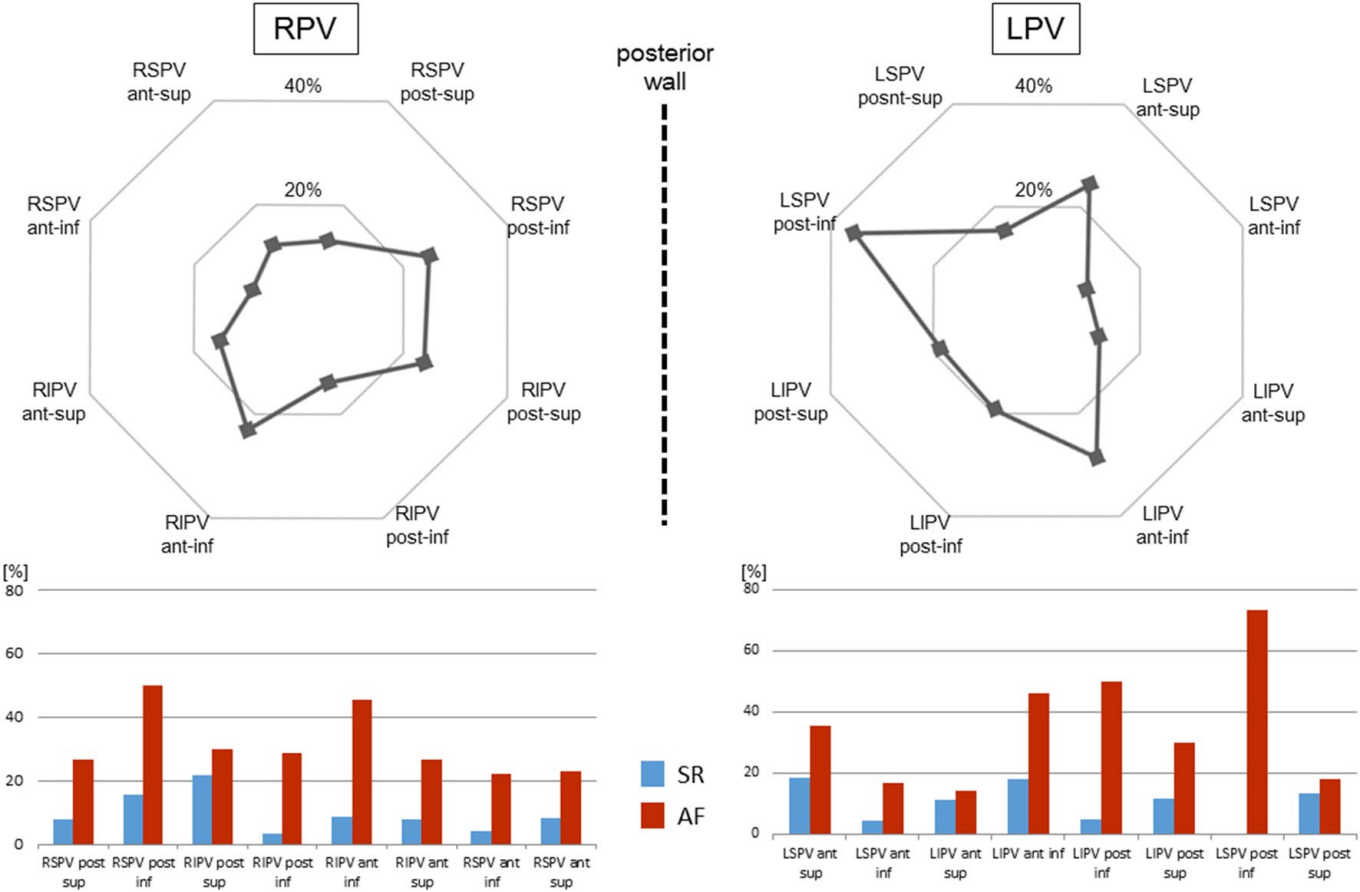
The percentage of points with constant contact according to anatomical location. *Upper* *part* the proportion of constant contact in all data points. Small percentage of the achievement of constant contact was shown in the ridge area (9.8 % at anterior-inferior of LSPV and 12.2 % at anterior-inferior of LIPV). *Lower part* the percentage of points with constant contact per each anatomical location according to atrial rhythm. Except for location at the posterior superior of LSPV (*p* = 0.18), the percentage of constant contact was significantly higher during AF than it was during SR (*p* < 0.05). The abbreviations used here are same as those used in Fig. 6

Average CF was generally higher at the right pulmonary veins (RPV) than it was at the left pulmonary veins (LPV). Average CF at the RPV was significantly higher compared with the LPV during both atrial rhythms (during SR 18.2 ± 11.7 g at the RPV vs. 16.4 ± 10.4 g at the LPV; *p* < 0.05, during AF 20.1 ± 14.4 g at the RPV vs. 14.1 ± 11.0 g at the LPV; *p* < 0.01). Lowest CF was observed at the LPV from anterior-inferior of LSPV (13.3 ± 8.8 g) and down to anterior-inferior of LIPV (12.0 ± 9.4 g). At this anatomical location (the LA ridge area), there was no significant difference of average CF between SR and AF (Fig. 6), but the proportion of constant contact was lower than in all other areas analyzed. The percentage of points with constant contact in the LA ridge area was significantly higher during AF than it was during SR (*p* < 0.01). The same holds true for the other anatomical locations mentioned here (Fig. 7).

At the anterior-inferior RIPV the highest average CF of all locations was achieved (25.7 ± 13.2 g). Average CF during AF was significantly higher than SR at the anterior-superior (18.1 ± 11.7 g during SR vs 25.9 ± 15.1 g during AF, *p* < 0.05) and anterior-inferior of RIPV (23.4 ± 11.5 g during SR vs 30.5 ± 15.5 g during AF, *p* < 0.05). Except for the anterior part of the RIPV, there was no significant difference of average CF between SR and AF in relation to the anatomical location (Fig. 6).

## Discussion

Our study has demonstrated that (1) average CF showed no significant difference between SR and AF; (2) regardless of the atrial rhythm, there was a high variability of average CF; (3) there was no significant difference in the ratio of either low contact or sufficient contact; however, the ratio of excessive contact was significantly higher during AF than during SR; (4) constant contact was more often achievable during AF than during SR; (5) there was no significant difference in the average CF between atrial rhythms according to the anatomical location except at the RIPV anterior area.

To the best of our knowledge, this study is the first systematic, prospective analysis of CF during different atrial rhythms (SR vs AF).

### Average CF and contact type according to atrial rhythm

Average CF varied widely during both atrial rhythms in the present study. We have demonstrated that there was no significant difference in average CF between atrial rhythms when CF information was blinded to the operators. Some clinical trials [3, 4] showed that average CF correlates with clinical outcome in patients with AF undergoing RF using CF-sensing catheters. We have shown here that low contact occurs during both atrial rhythms to the same degree (*p* = 0.18), and only 34 % points were categorized as sufficient contact during both atrial rhythms (34.6 % during SR, 34.2 % during AF, *p* = 0.51) when operators are blinded to CF information. On the other hand, excessive CF of more than 40 g is associated with potentially major complications, such as cardiac perforation or steam pop [10, 11]. In an animal model, a CF below 40 g did not result in any perforation [12]. In our study, more than 7 % points during AF came into excessive CF category, while just 4.2 % points during SR were categorized as an excessively high contact. The distribution of excessive contact was significantly higher during AF than during SR.

The results of the present study could be explained by the indication of two aspects, that is, (1) the difference of the atrial contractility and (2) the difference of indirect CF information.

Compared to the regular atrial contraction during SR, the atrial contractile function is diminished during AF due to very high frequent electrical activity. With no atrial contraction, stable catheter tissue contact might be more often achievable during AF than during SR. Accordingly, we could demonstrate that constant contact was more often achievable during AF than it was during SR although an average CF was not seen to be different between both rhythms. This result could be explained by the difference of atrial contractility between atrial rhythms.

The second factor which may influence tissue contact is the indirect information on CF which the operators have to rely on during the procedure. This study was performed with the operators blinded to the CF information. When operators have no access to direct and real-time CF information, the information about tissue contact which they do have is limited to indirect parameters. Fluoroscopic visualization of catheter tip or tactile feedback from catheter manipulation are assessed subjectively [13, 14]. Other conventional parameters such as impedance or amplitude of the local electrogram can be used as alternative markers of tissue contact. However, the accuracy of these indirect parameters has not been well validated and it has been postulated that they correlate poorly with tissue contact [10, 15–18]. As shown in a previous study [19], the amplitude or width of the local electrogram is quite different between SR and AF. Due to the very high frequent atrial electrical activity during AF, it is more difficult to recognize the local electrogram compared with that of SR. This difficulty in interpretation of catheter tip electrogram during AF may not only lead to insufficient contact but also has the potential of excessive contact when no CF information is available.

A previous study using CF sensing catheters in a contractile bench model demonstrated that constant contact produces the largest lesions in comparison with variable contact or intermittent contact [20]. In the present study we were able to show that stable tissue contact is more often achievable during AF, which might lead to larger and longer lasting lesions. This hypothesis has to be confirmed in future trials together with the utilization of larger sample sizes.

### CF during AF and SR according to anatomical location

Influences of the anatomical situation on CF were recently reported [3, 4, 6]. In these previous studies, average CF has been shown to be generally higher at the RPV than at the LPV. The present study demonstrated also a higher average CF at the RPV regardless of atrial rhythm. This study also showed that the lowest CF is at the LA ridge area. This result is also in accordance with previous studies [3, 4, 6]. No significant difference of average CF between atrial rhythms has been shown at the LA ridge area. This anatomical location is the most difficult location where one can achieve sufficient tissue contact due to complex anatomical situation, which is located between LPV and left atrial appendage. Inadequate CF leads to insufficient lesion and results in acute PV reconnection [5, 14]. It is well known that PV reconnection plays an important role in clinical recurrence of AF after RFCA of AF [21–23]. Low average CF and the small percentage of constant contact at the ridge area may explain the high rate of PV reconnection in this region [24]. The wall thickness of the LA ridge area has been shown to be an independent predictor of an AF recurrence [25]. At this area, CF information may yield an important additional value during both atrial rhythms.

At the anterior part of RIPV, the highest average CF of all positions was achieved. This location was the only area where the average CF showed any statistical difference between atrial rhythms (average CF was higher during AF than SR, *p* < 0.05). This area has a tendency to achieve high tissue contact. Previous studies have also shown high average CF in this area [4, 6]. Due to the close proximity to the transseptal puncture site, a more rigid fulcrum is used to achieve stability of the ablation catheter. Furthermore, maximum catheter deflection combined with a deflectable sheath may result in high CF when targeting the anterior part of RIPV. The anatomical relationship between ascending aorta and anterior LA is also considered to be a reason for high CF. When the ablation catheter is located directly beneath the ascending aorta, it exerts an external force against the LA wall and the ablation catheter [16]. In addition to these anatomical factors, the tendency of stable tissue contact and the difficulty of recognition of catheter tip electrogram during AF may have a potential of excessive contact when no CF information is provided. The present study suggests that particular caution should be taken in order to prevent excessive contact force when RFCA is performed at this area during AF. The real-time CF information to avoid excessive contact may potentially reduce the risk of major complications.

### Study limitations

There are several limitations of the study which should be acknowledged. First, this study was designed to assess the influence of atrial rhythm upon CF and has not attempted to indicate any clinical outcome. The impact of CF guided RFCA on the clinical outcome was recently reported [26, 27]. Although our objective was to evaluate the influence of atrial rhythm upon CF prospectively, the findings suggest that the additional CF information may bring an important value. Secondly, the procedures were performed under deep sedation rather than under general anesthesia. Although this is an acceptable mode of sedation during RFCA of AF, the differences of respiratory condition between patients may have added an uncontrolled variable to this study. Thirdly, not only was the sample volume relatively small, the study was performed in a single center. However, we were still able to find out the statistically meaningful differences with these relatively small numbers suggesting that atrial rhythm influences the CF. Larger, multicenter studies are required to confirm our initial results.

## Conclusion

Although average CF was not different between atrial rhythms, constant contact was more often achievable during AF than it was during SR. However, excessive CF also seems to occur more frequently during AF especially at the anterior part of RIPV during AF when no CF information is provided.
